# Dynamic changes in myocardial architecture after reperfused acute myocardial infarction (AMI): insights from the prospective REMI study (REmodeling after Myocardial Infarction)

**DOI:** 10.1186/1532-429X-16-S1-P223

**Published:** 2014-01-16

**Authors:** Lin Zhang, Olivier Huttin, Frédéric Moulin, Pierre-Yves Marie, Damien Mandry

**Affiliations:** 1 CHU Nancy, Nancy, France; 2 IADI U947, INSERM, Nancy, France; 3 Universite de Lorraine, Nancy, France

## Background

Following an acute infarction, a dynamic healing process initiates via extracellular substrate deposition and turnover, resulting in ongoing remodeling of infarct territory and global function change of left ventricle. Our prospective study was designed to investigate by CMR the dynamic changes in myocardial architecture at 2-4 days and 6 months following a reperfused AMI.

## Methods

66 consecutive patients who fulfilled inclusion criteria underwent two CMRs: 2-4 days (baseline) and 6 months (follow-up) after the AMI, comprising SSFP images for analysis of left ventricular functions and late gadolinium enhancement (LGE) MRI (0.1 mmol/Kg; DOTAREM^®^, GUERBET, Roissy, France) for infarct analysis using a threshold method. Of 132 CMRs, 6 (4 from baseline) were non-diagnostic owing to poor EKG triggering and/or inability of the patients to hold their breath; 5 patients without evidence of infarct scar by LGE were also excluded. Hence, 57 patients (50 male; 56.7 ± 13.2 yo) were eventually analyzed. Total infarct was determined as area with signal intensity (SI) above mean+5SD of remote myocardium and was further divided into peri-infarct zone (PIZ) and infarct core by introducing a 7SD threshold; microvascular obstruction was manually included into the core; a core to PIZ ratio(C/PIZ) was calculated and a transmurality score was computed using a 5-point scale on the 16-segment model derived from the AHA model. Paired t-test was used for comparison between the two MRI and Pearson's correlation coefficients were calculated to assess prediction of LV parameters.

## Results

14 patients (25%) had an AMI in the LAD territory; 18 patients (31.6%) presented microvascular obstruction on LGE images at baseline. Dynamic changes of infarct indices and LV functional parameters are detailed in table 1; LVEF improved significantly (p < 0.0001), owing to a decrease of LVESV (p < 0.05), possibly related to stunning at subacute phase; the increase of LVEDV remained mild, possibly indicative that most of LV remodeling occurred prior to the baseline MRI. Total infarct, core and PIZ mass all diminished significantly (p < 0.0001 for all). Both C/PIZ and transmurality score remained identical. Both total scar and infarct core masses at baseline were predictive of LVEF, LVEDV and LVESV at follow-up (table 2), but not of the change in LV functional parameters.

**Table 1:** Infarct index and LV functional parameters at baseline and follow-up.

	Baseline (N = 57)	Follow-up (N = 57)	Change	%Change	P value
LV mass, g	97.2 ± 19.1	92.9 ± 20.4	-4.2 ± 15.2	-3.7 ± 15.8	<0.05

LVEF,%	43.0 ± 6.7	49.4 ± 7.7	6.4 ± 5.8	15.7 ± 15.3	<0.0001

LVEDV, mL	177.4 ± 30.4	183.0 ± 36.7	5.6 ± 22.1	3.4 ± 12.7	NS (0.061)

LVESV, mL	100.3 ± 22.7	94.4 ± 30.0	-5.9 ± 19.5	-6.2 ± 18.5	<0.05

Infarct mass, g	25.4 ± 11.8	17.0 ± 7.9	-8.5 ± 8.0	-30.6 ± 20.1	<0.0001

Infarct mass, % of LV mass	25.4 ± 8.8	17.8 ± 6.3	-7.6 ± 6.3	-27.5 ± 20.0	<0.0001

Core mass, g	12.3 ± 6.0	10.5 ± 5.7	-2.8 ± 5.3	-17.2 ± 41.3	<0.0001

Core mass, % of LV mass	13.3 ± 4.6	10.8 ± 4.8	-2.4 ± 4.6	-14.6 ± 37.4	<0.0001

PIZ mass, g	9.6 ± 4.5	6.5 ± 2.9	-3.2 ± 3.5	-26.6 ± 26.3	<0.0001

PIZ mass, % of LV mass	9.7 ± 3.6	6.9 ± 2.7	-2.7 ± 3.2	-22.8 ± 28.2	<0.0001

Core/PIZ	1.6 ± 0.8	1.7 ± 0.8	0.1 ± 1.0	28.4 ± 80.5	NS(0.405)

Transmurality score	1.30 ± 0.31	1.21 ± 0.43	-0.1 ± 0.45	-3.53 ± 35.68	NS(0.118)

LV indicates left ventricular; LVEF, LV ejection fraction; LVEDV, LV end-diastolic volume; LVESV, LV end-systolic volume; PIZ, peri-infarct zone; NS, no significant

**Table 2:** Pearson's correlation coefficients between infarct indices and LV remodeling.

	LVEF_2_,%	ΔLVEF,%	LVEDV_2_, mL	ΔLVEDV, mL	LVESV_2_, mL	ΔLVESV, mL
Infarct mass_1_, g	-0.426**	-0.096	0.598**	0.228	0.601**	0.207

Infarct mass_2_, g	-0.486**	-0.198	0.615**	0.420**	0.625**	0.358**

Core mass_1_, g	-0.408**	-0.157	0.500**	0.137	0.518**	0.189

Core mass_2_, g	-0.497**	-0.248	0.561**	0.491**	0.598**	0.440**

PIZ mass_1_, g	-0.144	0.08	0.519**	0.111	0.436**	0.100

PIZ mass_2_, g	-0.341**	-0.05	0.561**	0.175	0.518**	0.108

Core/PIZ_1_	-0.23	-0.167	-0.109	-0.008	0.012	0.053

Core/PIZ_2_	-0.291*	-0.191	0.129	0.415**	0.209	0.359**

%change Core/PIZ	-0.198	-0.04	0.281*	0.409**	0.299*	0.319*

Transmurality score_1_	-0.274	0.011	0.362**	0.123	0.331*	0.007

Transmurality score_2_	-0.160	0.089	0.238	0.205	0.204	0.000

*p < 0.05; **p < 0.01; subscript 1 and 2 indicate baseline and follow-up data, respectively.

## Conclusions

Following reperfused AMI, our study demonstrated a significant improvement in LVEF, related to an improved contractility due to stunning in the subacute phase. Myocardial damage characteristics by LGE showed a decrease of infarct size, owing to resorption of myocardial edema, in the same amount between its two components, core and PIZ.

## Funding

Grant from the French Ministry of Health Support from Laboratoire Guerbet, Roissy, France: supply of contrast medium

